# Chromium removal from tannery wastewaters with a strong cation exchange resin and species analysis of chromium by MINEQL+ 

**DOI:** 10.1038/s41598-022-14423-3

**Published:** 2022-06-10

**Authors:** Sevgi Kocaoba, Gulten Cetin, Goksel Akcin

**Affiliations:** Department of Chemistry, Faculty of Art and Science, Yildiz Technical University, Davutpasa, Istanbul, Turkey

**Keywords:** Environmental sciences, Natural hazards, Chemistry

## Abstract

Chromium (III) salts are highly applied for tanning purpose in tannery industries. The purpose of this study was removal and recovery of chromium(III) from tannery wastewater with a strong cation exchange resin. For this purpose, Amberlite 252 ZU was chosen as a strong cation exchange resin. In the first part of this study, The MINEQL+ computer program was applied depending on the optimum concentration and pH for determining Cr species in aqueous solutions. The second part of the work consists of measuring the exchange equilibrium of H+ ions and Cr(III) ions. Therefore, solutions containing fixed amounts of chromium were brought into contact with different amounts of resins. The evaluation of the obtained equilibrium parameters was done by surface complexing theory. Retention and regeneration steps were successfully performed in the column without any significant change up to 10 cycles. Efficiency was between 90 and 98% in removal studies, and between 81 and 92% in recovery studies. The results showed that a strong cation exchange resin Amberlite 252 ZU can be successfully used for chromium removal and recovery.

## Introduction

Environmental pollution and reducing its damages has been a subject that has been studied for a long time. Removal problems of pollutants from wastewater are growing with rapid industrialization. Heavy metals, oils, suspended solids, organic substances and other similar pollutants that have toxic effects for many organisms spread to the environment from the wastes of industries such as leather, textile, paint and printing, electroplating applications, food industry, wood preservative and miscellaneous etc^1–7^. Untreated wastewater from such industries causes an increase in chromium content in the environment and groundwater^8–15^. Chromium toxicity limits are 28–80 mg/L for fish and 0.05 mg/L for drinking water. The level of chromium that people can get from daily food is 0.05–0.2 mg/day. In addition, chromium has toxic and carcinogenic effects, as well as a tendency to accumulate in living organisms. It is predicted that excess chromium in the body can damage the kidneys and increase the risk of lung and stomach cancer.

Chromium is generally found in two species in different environmental samples. Cr(III) and Cr(VI), which are predominantly two oxidation states, show significant differences in their biological and toxicological behavior. Cr(III) and (VI) are inorganic chromium species in water. While Cr(VI) is known to be more toxic and carcinogenic, Cr(III) specie is necessary for maintaining glucose tolerance in humans. Due to these harmful properties and high solubility of Cr(VI), environmental protection studies have focused on this type of chromium. On the contrary, the toxicity of Cr(III) compounds is lower than Cr(VI) and can be easily precipitated in neutral or basic conditions. Therefore, the traditional treatment method of chromium-containing wastewater is to reduce Cr(VI) to Cr(III) and precipitate as Cr_2_O_3_⋅xH_2_O at high pH values^16–21^.

Leather tanning is one of the most polluting agro-industrial sources and industrial activity holds an important place in Turkey. Large amounts of tannery wastewater containing chrome salts and other pollutants are poured into open areas, agricultural lands and various water sources, causing great pollution of the soil, water and ecosystem. Due to their toxicity, the maximum permitted metal levels in wastewater, even at low concentrations, are regulated by separate legislation in each country. All tanneries containing 1500 mg/L or more of Cr(III) ions must take effective measures to treat waste chrome tanning solutions. However, the high economic and environmental costs associated with greater land disposal make it difficult for many tanneries to effectively treat their wastewater. There is a need for an effective and more economical method of removing chromium from the leather industry wastewater, including the recovery and reuse of chromium. This is important both in terms of protecting the environment and reducing raw material costs. Various conventional precipitation and ion exchange processes have been developed for the removal and recovery of chromium and other heavy metals from wastewater. New methods are being explored to effectively improve existing technology for chromium removal and recovery. However, more traditional and old methods for wastewater treatment are still being used, which can also release more waste to the environment^22–31^.

The precipitation method is generally preferred for removal of chromium from wastewaters. According to this method, Cr(VI) is reduced to Cr(III) and precipitated in the form of hydroxide. For reduction, the pH range should be brought to the 2–3 range. After this step, Cr(VI) is reduced to Cr(III) with a reducing agent (SO_2_, NaHSO_3_, Na_2_S_2_O_3_, Na_2_SO_3_). Neutralization in the second step and precipitation with OH^−^ is provided^32–36^. Other removal methods are electrochemical precipitation, adsorption, ion exchange, solvent extraction, membrane and foam separation, reverse osmosis, biosorption etc^37–46^.

Ion exchange method is a very effective, inexpensive and highly preferred method for removing and recovering heavy metals from liquid wastes. As we have seen in our and other previous studies that strong cation exchange resins are very effective for removal and recovery of chromium. Therefore, Amberlite 252, as a strong cation exchange resin, was used in this study. The principle of the study involves the removal of Cr(III) ions from tannery waste water with the help of a strong acidic ion exchange resin in hydrogen form. It is well known from previous studies that chromium species are easily removed in this way. The removed chromium(III) values were changed between 80 and 98% ^22,47–52^.

The regeneration method used to recover chromium species from resins is extremely important because concentrated acids or salt solutions traditionally used in the recovery of other metals cannot be made very satisfactorily due to the strong binding of trivalent chromium ions. Therefore, as recommended in the literature^37,47^, it has been found that the application of H_2_O_2_ in alkaline medium during the regeneration process is very useful to solve this problem. In this way, Cr(III) species are converted into chromate ions. The resin is loaded with sodium ions in alkaline medium. In the last step, it is re-converted to H^+^ form by means of sulfuric acid.

With the method used in this study, not only chromium removal is performed, but also the amount of waste is reduced and the consumption of raw materials is minimized by recovering the consumed chromium. The investigations have occurred with batch studies. In batch studies, equilibrium experiments were done for determination of exchange equilibria (exchange Cr^3+^ for H^+^). The evaluation of the obtained equilibrium parameters was done by surface complexing theory. In addition, The MINEQL+ computer program was applied depending on the optimum concentration and pH for determining chromium species in aqueous solutions. As a result, it has been found that chromium can be effectively removed from tannery wastewater with Amberlite 252 ZU, a strong cation exchange resin. In addition, it has been observed that chromium, a precious metal, can be recovered with the applied regeneration process. Therefore, chromium removal and recovery was achieved successfully with the chosen method. With the MINTEQ program applied, the species formed were determined in order to find the most suitable optimum conditions in the pH and concentration ranges studied.

## Materials and methods

### Materials

In this study, Amberlite 252 ZU (strong acidic cation exchange resin) was used for the determination of the exchange equilibria of Cr(III) for H^+^ by using chromium chloride solutions in acidic pH. The properties of Amberlite 252 ZU are given in Table 1. Analytical grade chemicals, CrCl_3_.6 H_2_O HCl, HNO_3_, NaCl, NaOH (Merck, Germany) were used in the preparation and application of the resins to the studies. Analytical grade chemicals, H_2_O_2_, NaOH, H_2_SO_4_ (Merck, Germany) were used for regeneration of the resin. All solutions used for the resin preparation, ion exchange and resin regeneration were prepared freshly from double distilled water.

**Table 1:** Properties of the resin.

Data	Ion exchange resin (Amberlite 252 ZU)
Matrix	Styrol-DVB
Functional group	Sulphonic acid
Particle size	0.6–0.8 mm
Max. temp	100 °C
pH range	0–14
Total capacity	≥ 1.8 eq/L

### Preparation of the resin

Amberlite 252 ZU ion exchange resin was pretreated in a column by three treatment cycles with 1 M HCl and a mixture of 1 M NaOH and 1 M NaCl to remove impurities that trapped in its matrix during manufacture. The ion exchange resin was then converted to H+ form with 1 M HCl. The resins to be used for equilibrium experiments were centrifuged before weighing to remove water attached to the outer surface of the particles.

### Determination of total capacity of the resin

It was determined in the batch system. 1 g of resin was weighed into erlenmeyer flasks and agitated by a constant volume of solution (100 mL, 0.1 M NaOH solution) for 1 day at room temperature (20 ± 10 °C) with shaking until equilibrium was achieved. The initial and final amounts of sodium were measured by atomic absorption spectrophotometry (AAS) to determine the total capacity of the resin. At the same time, capacity studies were carried out ten times by repeating the retention and regeneration steps to determine the effectiveness of the column. The exchange capacities of resin were calculated with the following equation;

1 $${\text{Q }} = \, \left( {{\text{C}}_{{{\text{initial}}}} {-}{\text{ C}}_{{{\text{final}}}} } \right){\text{ V }}/{\text{ m}}$$

C_initial_ is the initial concentration of sodium, C_final_ is the final concentration of sodium (meq/L), V is the solution volume (L) and m: resin amount (g).

The results are given in Table 2.

**Table 2:** Exchange capacities of Amberlite 252 ZU.

Cycle	Capacity (meq/g)
Beginning	2.82 meq/g
5th cycle	2.56 meq/g
10th cycle	2.15 meq/g

### Speciation of chromium

**Figure 1:**
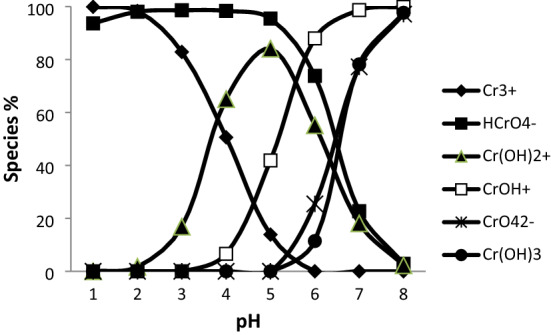
Species % for chromium depends on pH.

Chromium species were investigated with respect to pH for Cr-H_2_O systems with MINEQL+ version 3.01 speciation working program. Information about the MINEQL+ computer program is given in the literature^53^. According to the parameters effective in adsorption such as concentration and pH, % concentrations of the chromium species were found in the pHs examined using the MINEQL+ program. The speciation diagram has given in Fig. 1.

### Equilibrium experiments

#### Determination of exchange equilibria [exchange Cr^3+^ for H^+^]

**Figure 2:**
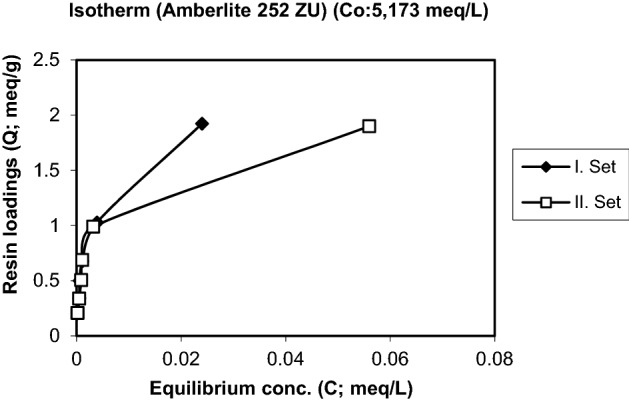
Isotherm of the uptake of Cr^3+^ by the resin Amberlite 252 ZU (Initial conc. and pH: 5.173 meq/L, 3.18).

**Figure 3:**
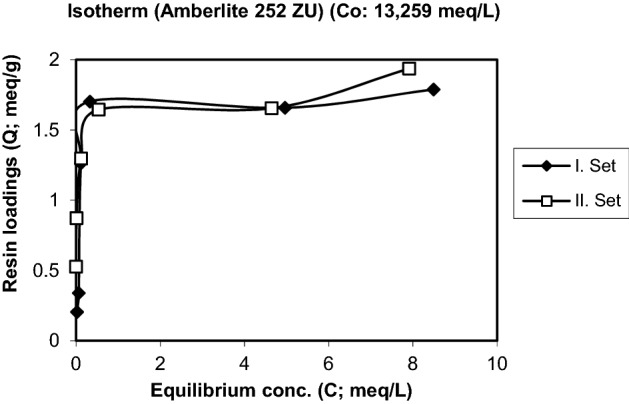
Isotherm of the uptake of Cr^3+^ by the resin Amberlite 252 ZU (Initial conc. and pH: 13.259 meq/L, 3.15).

**Figure 4:**
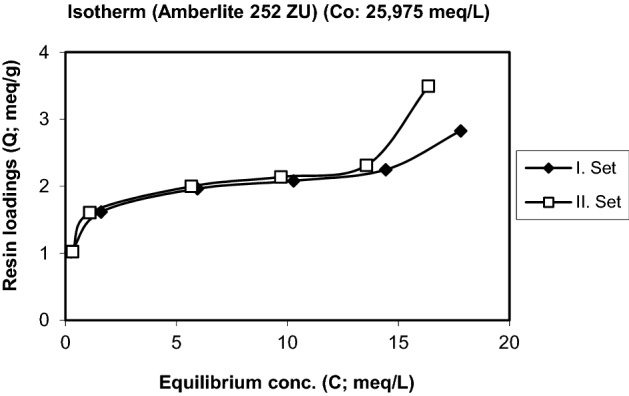
Isotherm of the uptake of Cr^3+^ by the resin Amberlite 252 ZU (Initial conc. and pH: 25.975 meq/L, 2.92).

To investigate the exchange equilibria of hydrogen ions with Cr(III) ions, 0.5, 1, 1.5, 2, 3, 5 g resin samples were weighed into flasks with lids. In a shaker, it was contacted with 200 mL volume of chromium solutions at concentrations of 5.173 meq/L, 13.259 meq/L and 25.975 meq/L at room temperature (20 ± 10 °C) for 5 days. Since pH is an important parameter in equilibrium studies, pH values were changed in each experiment series. The initial and final equilibrium pH values were measured with an electrode and a pH meter. The surface complexation theory was applied for evaluation of experimental results and prediction of equilibria. According to this theory, the surfaces of ion exchange resins are considered to be a planar surface on which functional groups (ions) are evenly distributed. Surface loads are produced by separating or protonating surface groups. As a result, it can be thought that the protons are held directly on the surface. Most of other ions are found in Stern layers parallel to the surface and at different distances from the surface. The remaining opposite and same charged ions are distributed throughout the normally negligible scattered layer. In the evaluation of equilibrium parameters, generalized separation factors are determined from equilibrium concentrations and resin charges, and the sum of resin loadings (algebraic) is plotted against dimensionless resin loadings. The isotherms are given Figs. 2, 3 and 4.

### Preparation of samples for analysis

Samples taken from the liquid phase were filtered through 0.45 µm Millipore filters and samples with high chromium concentrations were diluted to appropriate determination concentrations. All samples were acidified 1% with absolute nitric acid solution before determination. Chromium concentrations were measured by atomic absorption spectrophotometry.

### Application of surface complexation theory to data

The retention of ions on charged surfaces can also be explained by the surface complexing model. The surface charges of a strongly acidic ion exchange resin are created by dissociation of surface groups. Negative surface potentials are formed during the dissociation of sulfonic groups in the structure of a strongly acidic cation exchange resin. Therefore, while the same charged ions are repelled, the counter-charged ions generate attractive forces. As a result of retaining counter-charged ions in functional groups, a fractional reduction in surface potential occurs. According to this theory, each counter-charged ion is assumed to be at a certain distance from the surface. This causes the formation of regular double or Stern layers. Ion pairs formed between surface groups and counter-charged ions in the regular layer are defined as surface complexes. Excessive charges on the surface are balanced with counter-charged ions in dispersed layer containing co-charged ions. As a result, the surface potential is continuously reduced by the distance from the surface until it is zero in the liquid phase. In fact, prototype reactions dominate the balance between solid and liquid phases, making protons effective in determining surface potential^54–61^.

In systems with CrCl_3_ and pH less than 3, the exchange of Cr(III) ions with H^+^ ions in the resin structure takes place as follows;

$$\overline{{R - SO_{3}^{ - } \,H^{ + } }} \; + \;(Cr^{3 + } )\quad \Leftrightarrow \quad \overline{{R - SO_{3}^{ - } \,(Cr^{3 + } )}} \; + \;\,H^{ + }$$

When we applied surface complexation model to experimental data, it is based on the assumption of a specific series of Stern layers of counterions and on the introduction of the respective “Generalized Separation Factor”. Considering their local equilibria, it can be said that hydrogen ions are adsorbed on a Stern layer closer to the surface than chromium ions, and the following expression is simplified as given in the literature ^54,56^ for the calculation of the generalized separation factor;

2 $$\log \,Q_{Cr}^{H} = \log \frac{{y(Cr).C(H)^{3} }}{{y(H)^{3} .C(Cr)}}$$

*y*(*Cr*) is the dimensionless loading of chromium in resin phase, *y*(*H*) is the dimensionless loading of hydrogen in resin phase, log *Q*^*H*^_*Cr*_ is the logarithm of the generalized separation factor, *C(Cr)* is the liquid phase chromium ions concentration (meq/L), *C(H)* is the liquid phase hydrogen ions concentration (meq/L).

The respective numerical values of the generalized separation factor were determined from experimental data. The equilibrium parameters result from the linear relationship

3 $$\log Q_{Cr}^{H} = \log K_{Cr}^{H} + m(H,Cr).y(Cr)$$

From this plot the two equilibrium parameters *K*^*H*^_*Cr*_ and m(H,Cr) can be obtained. The slope m(H,Cr) contains the electric capacitance of the capacitor formed by the layers of H and Cr ions. *y*(*Cr*) being obtained from:

4 $$y\left( {Cr} \right) \, = {\text{Q}}_{{{\text{Cr}}}} /{\text{ Q}}_{{{\text{max}}}}$$

and *y*(*H*) was obtained from the measured pH values. Therefore, for local equilibria, logarithmic equilibrium parameters (generalized separation factors) can be obtained by measuring the pH values and equilibrium concentrations. As has been shown theoretically the parameter m, being the slope of the linear relationship (Eq. 3) has to be ≥ 0. Therefore, if evaluation leads to negative values the assumed sequence of Stern layers was incorrect and has to reversed. For the reverse sequence the separation factor is log Q^Cr^_H_ and has to be calculated from an expression with the inverse quotient appearing in the logarithm. Furthermore, the equilibrium constants are log K^Cr^_H_ and m(Cr, H).

For evaluation of equilibrium parameters the generalized separation factors determined from each of the samples have to be plotted vs. the respective dimensionless resin loading with the counterion which is located farther away from the surface.

**Figure 5:**
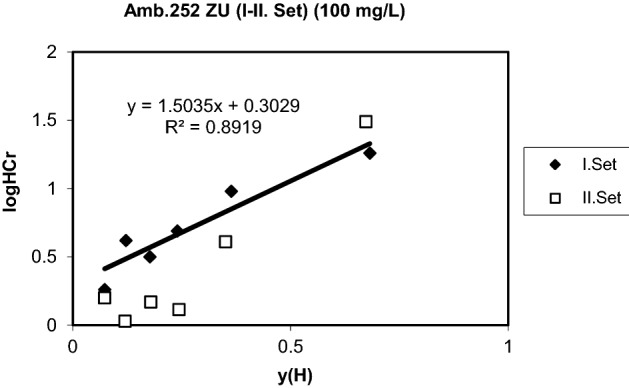
Generalized separation factor for the exchange of H^+^ for Cr^3+^ (Initial conc. and pH: 5.173 meq/L, 3.18).

**Figure 6:**
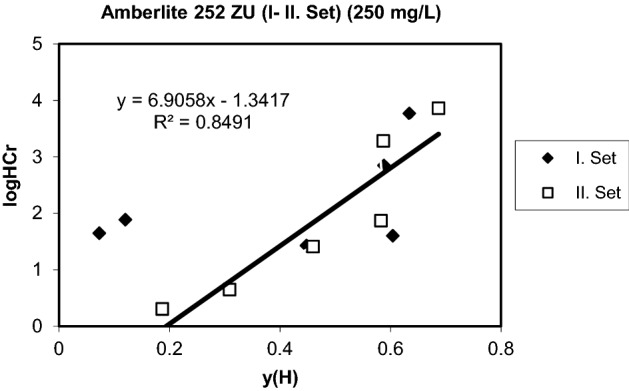
Generalized separation factor for the exchange of H^+^ for Cr^3+^ (Initial conc.and pH: 13,259 meq/L, 3.15).

**Figure 7:**
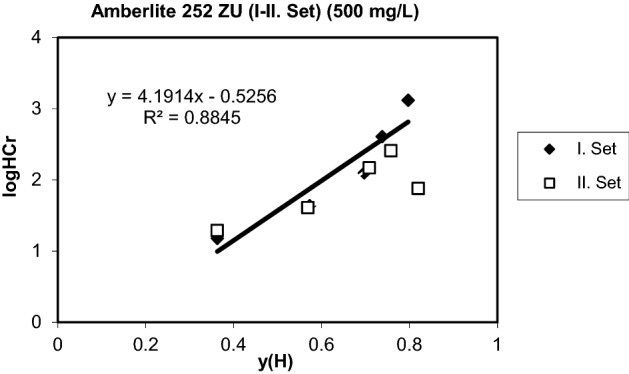
Generalized separation factor for the exchange of H^+^ for Cr^3+^ (Initial conc.and pH: 25.975 meq/L, 2.92).

The results are given in Figs. 5, 6 and 7. Equilibrium parameters of the exchange for protons are given in Table 3.

**Table 3:** Equilibrium parameters of the exchange for protons (Amberlite 252 ZU).

Co (mg/L)	logQHCr	m (H,Cr)
100	1.5035	0.3029
250	6.9058	1.3417
500	4.1914	0.5256

## Results and discussion

### Speciation of chromium

Speciation was done in studying pH with MINEQL+ computer program. The % concentrations and pH values of the chromium species found in the medium were found with this computer program. The speciation diagram has given in Fig. 1 shows the dispersion of the chromium species at different pH values in aqueous solutions. Simulations based on the MINEQL+ program largely depend on the composition of the liquid phase and the pH of the solution.

The determined study pH’s and concentration values (20 mg/L) were chosen to compare experimental and computerized results.

According to the chromium speciation diagram for pH, the predominant species below 3 is Cr^3+^ , between 4–5, Cr(OH)^2+^ and after pH 6, the predominant species is Cr(OH)3, because of precipitation Cr(III) as Cr(OH)3. It was not preferred to work after pH 6 in batch studies due to the precipitation of chromium. At pH 4, the Cr^3+^ and Cr(OH)^2+^ species are present in approximate distribution of around 30 and 65%. At pH 5, the Cr(OH)^2+^ species dominates, accounting for nearly 84% of the chromium present with other major form as Cr(OH)^+2^ accounting for around 4%. These results are similar to the speciation diagram of reference for chromium complexes present in aqueous solution^47^. Looking at the distribution of the species between pH 3 and 4, it clearly shows that the Cr (OH)^2+^ complex with Cr^3+^ is selectively adsorbed into the resin, but between pH values of 4 and 6, Cr(III) is retained principally as Cr(OH)2, although the Cr(OH)^+2^ complex is also retained. In addition, Cr^3+^, Cr(OH)^2+^, Cr(OH)^+2^, Cr(OH)3 species were found with a maximum retention at pH 5. At this pH, the Cr(OH)^2+^ specie predominates approximately 84%. Maximum retention of chromium is shown at pH 5, but since we prefer to work in Cr^3+^ form and the dominant species is Cr^3+^ at pH: 3, all studies have been done at pH: 3.

### Exchange isotherms

Isotherms of the exchange of Cr^3+^ for H^+^ are plotted in Figs. 2, 3 and 4 for three different initial concentrations of Cr^3+^. Figure 2 shows that the resin loading increases strongly in a narrow concentration range region. At higher initial concentrations the typical isotherms for a strongly preferred species develop with a sharp increase at small concentrations and a more or less flat further development. The maximum loadings obtained are in the range of 2 to 3.5 meq/g.

### Equilibrium parameters

Equilibrium parameters were determined by calculating the generalized separation factor as explained before. When the data were evaluated, it showed that a positive slope generalized separation factor can only be found assuming that chromium ions are in the inner layer and hydrogen ions are closer to the surface than chromium ions. This situation is similar to the previous equilibrium studies with strong cation exchange resins ^48,54,62–66^.

It has been shown that more counter ions in Stern layers are fixed with increasing log K values, and at stronger dissociation values, the counter ions are increasingly found in scattered layers. The straight line from the graphs shows that the metal ions are slightly farther away from the surface of the Stern layer, while the protons have a positive slope indicating that they are exposed to certain interactions with functional regions and are directly in the plane of the surface. Similar comments have been made in some studies where the surface complex theory was previously applied ^56,63,64,67–70^. The results are given in Figs. 5, 6 and 7 for two set of experiments. The two equilibrium parameters required for the evaluation of the ion exchange equilibrium, $\log Q_{Cr}^{H}$ and m(H,Cr) were found from the graphs and are given in Table 3. The preference for chromium leads to positive values of logQ^H^_Cr_. The slope m(H,Cr) contains the electrical capacitance of the capacitor, which consists of layers of H and Cr ions. Both of these parameters must be constant to hold a metal ion by a particular sorbent and can be used for estimation of multicomponent equilibrium. The lower logQ^H^_Me_ value, the more preferable is the metal species (Me) by the sorbent. The greater value of m(H,Me), the greater distance of Stern layers of metal ion from the surface of the sorbent. It is one of the advantages of the surface complexation theory that the parameters are independent of the total concentration. The surface complexation model provides a perfect definition and prediction of the counter equilibria by any type of ion exchange resins. It also facilitates accurate estimation of ion dispersions in resin and liquid phases^26,65,71–75^. Linear correlations were obtained in some cases with Amberlite 252 ZU resin. This may be due to the neglect of counterions in the diffused layer. This region is relatively larger than other resin types for strong acidic exchange resins. Thus, it can be concluded that the dispersion of counterions in the diffuse layer is minimum. This has shown that strong coordination complexes between divalent and trivalent ions and ion exchange regions are well formed, as can be seen from previous studies^48,56,63–65,76–80^.

The removal of chromium with the strong acidic exchange resin (Amberlite 252 ZU) used in this study was found to be very successful.

## Conclusions

The MINEQL+ computer program was applied depending on the optimum concentration and pH for determining chromium species in aqueous solutions. According to the chromium speciation diagram for pH, the predominant species below 3 is Cr^3+^ , between 4–5, Cr(OH)^2+^ and after pH 6, the predominant species is Cr(OH)3, because of precipitation Cr(III) as Cr(OH)3. In addition, Cr^3+^ , Cr(OH)^2+^ , Cr(OH)^+2^, Cr(OH)3 species were found with a maximum retention at pH 5. At this pH, the Cr(OH)^2+^ specie predominates approximately 84%. Maximum retention of chromium is shown at pH 5, but in order to preserve the trivalent chromium form studied in tanneries, we preferred to work at pH 3, where Cr^3+^ is dominant.

Equilibrium parameters for Amberlite 252 ZU were obtained from a series of binary experiments. In these sets, all experimental conditions were the same and were used to find the equilibrium from a theoretical basis. In most cases, a good agreement has been found between predicted and experimental data. The surface complexation model provided a perfect definition and prediction of counterion equilibria with Amberlite 252 ZU and facilitated accurate prediction of dispersion in resin and liquid phases. It was also found that chromium had a higher adsorption efficiency with the selected resin. Retention and regeneration steps were successfully performed in the column without any significant change up to 10 cycles. Efficiency was between 90 and 98% in removal studies, and between 81 and 92% in recovery studies.

The results showed that Amberlite 252 ZU, a strong cation exchange resin, can be successfully applied for removal and recovery of chromium.

## Data Availability

All data generated or analysed during this study are included in this published article.
